# A population-based cross-sectional study of colorectal cancer screening practices of first-degree relatives of colorectal cancer patients

**DOI:** 10.1186/1471-2407-13-13

**Published:** 2013-01-10

**Authors:** Ryan J Courtney, Christine L Paul, Mariko L Carey, Robert W Sanson-Fisher, Finlay A Macrae, Catherine D’Este, David Hill, Daniel Barker, Jody Simmons

**Affiliations:** 1 Priority Research Centre for Health Behaviour, School of Medicine and Public Health, Faculty of Health, University of Newcastle, Callaghan, Australia; 2 Department of Colorectal Medicine and Genetics, The Royal Melbourne Hospital, Melbourne, Australia; 3 The Centre for Clinical Epidemiology and Biostatistics, Faculty of Health, University of Newcastle, Callaghan, Australia; 4 Hunter Medical Research Institute, University of Newcastle, Callaghan, Australia; 5 Professorial Fellow, University of Melbourne, Melbourne, Australia; 6 Centre for Behavioural Research in Cancer, Cancer Council Victoria, Melbourne, Australia; 7 Priority Research Centre for Health Behaviour, University of Newcastle, Room 269a, Level 2, David Maddison Building, Callaghan, 2308, Australia

**Keywords:** Colorectal cancer, Screening, Prevention, Early detection, Family history

## Abstract

**Background:**

The aim of this study was to determine the proportions and predictors of first-degree relatives (FDRs) of colorectal cancer (CRC) patients (i) ever receiving any CRC testing and (ii) receiving CRC screening in accordance with CRC screening guidelines.

**Methods:**

Colorectal cancer patients and their FDRs were recruited through the population-based Victorian Cancer Registry, Victoria, Australia. Seven hundred and seven FDRs completed telephone interviews. Of these, 405 FDRs were deemed asymptomatic and eligible for analysis.

**Results:**

Sixty-nine percent of FDRs had ever received any CRC testing. First-degree relatives of older age, those with private health insurance, siblings and FDRs who had ever been asked about family history of CRC by a doctor were significantly more likely than their counterparts to have ever received CRC testing. Twenty-five percent of FDRs “at or slightly above average risk” were adherent to CRC screening guidelines. For this group, adherence to guideline-recommended screening was significantly more likely to occur for male FDRs and those with a higher level of education. For persons at “moderately increased risk” and “potentially high risk”, 47% and 49% respectively adhered to CRC screening guidelines. For this group, guideline-recommended screening was significantly more likely to occur for FDRs who were living in metropolitan areas, siblings, those married or partnered and those ever asked about family history of CRC.

**Conclusions:**

A significant level of non-compliance with screening guidelines was evident among FDRs. Improved CRC screening in accordance with guidelines and effective systematic interventions to increase screening rates among population groups experiencing inequality are needed.

**Trial Registration:**

Australian and New Zealand Clinical Trial Registry: ACTRN12609000628246

## Background

Worldwide, colorectal cancer (CRC) is diagnosed in over one million persons annually and is the fourth leading cause of cancer death [1]. Staging of disease at diagnosis is a critical factor affecting survival. When discovered early, CRC is highly treatable, with a relative five-year survival rate of 90% for localised CRC [2]. Several randomised controlled trials have demonstrated that CRC mortality can be reduced by 15% to 33% through Faecal Occult Blood Test (FOBT) screening, [3-6] with fewer advanced CRCs detected, compared with patients presenting with symptoms, in population-based screening [7]. Although the use of colonoscopy to detect right-sided CRCs is under debate, [8,9] case control and cohort studies of colonoscopy screening suggest a CRC mortality reduction ranging from 60% to 76% [10] and incidence reduction of 76% to 90% [11].

Approximately 15% to 25% of persons who develop CRC will have a first-degree relative (FDR), i.e. a parent, sibling or child, also affected by the disease [12,13]. Persons with one FDR diagnosed under the age of 55 years or with two FDRs diagnosed at any age have a three- to six-fold increased risk of developing CRC [14]. The relative risk of developing CRC is further increased where a known genetic mutation has been identified [15]. For persons where a known genetic mutation has been identified, both earlier onset of CRC and much higher risk are apparent. Given the increased risk imposed on FDRs of CRC patients, screening for CRC assumes major importance. Screening strategies targeting FDRs of affected cases could contribute to the prevention or early detection of 15% to 20% of CRCs [16,17]. Healthcare authorities and professional societies have published guidelines for the appropriate screening of FDRs of persons affected with CRC [18-20]. International approaches to risk classification vary slightly, but all follow the same pattern, with risk level determined by the number and type of relatives diagnosed (i.e. first- or second-degree), the age at diagnosis and the presence of other high-risk features, i.e. mutation status for cancer-predisposing genes if present in the family [18-20]. Screening guidelines for persons at higher risk generally recommend additional types of testing (e.g. colonoscopy rather than, or in addition to, FOBT), more frequent testing and commencement of testing at an earlier age, compared with their average risk counterparts [18-20]. Australian National Health and Medical Research Council (NHMRC) guidelines recommend that asymptomatic persons “at or slightly above average risk” commence screening at the age of 50 years and receive FOBT screening every two years or consider sigmoidoscopy (preferably flexible) every five years [18]. In Australia, [18] contrary to other international guidelines, [19,20] colonoscopy screening is endorsed only for asymptomatic persons at higher levels of risk (i.e. “moderately increased risk” or “potentially high risk”). For persons at “moderately increased risk”, colonoscopy is endorsed every five years starting at age 50 years or at ten years earlier than first diagnosis in the family, whichever comes first [18]. Endoscopy screening for persons at “potentially high risk” is recommended at least on a five-yearly basis in the Australian guidelines. However, age at screening commencement, test type and repeat testing interval are dependent on the type of family-specific mutation identified [18].

Despite the elevated risk associated with having a family history of CRC, the little available evidence suggests that adherence to recommended screening for FDRs of CRC patients is low [18]. While FDRs of CRC patients are more likely to be screened, compared with those without a family history of CRC, [21-23] screening compliance for this group is sub-optimal, at between 21% and 78% [16,21,24-27]. Scant literature exists related to screening participation in terms of published screening guidelines and across level of risk [28-31]. Further, relatively little is known about the factors associated with FDRs of CRC patients’ guideline-recommended screening compliance [21]. The aim of this study was to examine among FDRs of persons diagnosed with CRC and at each level of risk (“at or slightly above average risk”, “moderately increased risk” and “potentially high risk”), the proportions (i) ever receiving any CRC testing in their lifetime and (ii) screened in accordance with Australian CRC screening guidelines.

The individual- and provider-level factors associated with FDRs ever receiving CRC testing and guideline-recommended screening were also evaluated.

## Methods

### Setting and design

Index cases (i.e. persons diagnosed with CRC) and their FDRs were recruited through the population-based Victorian Cancer Registry (VCR), Victoria, Australia between 2009 and 2011. Human research ethics approval was obtained from The University of Newcastle and The Cancer Council Victoria.

### Procedure

**Figure 1:**
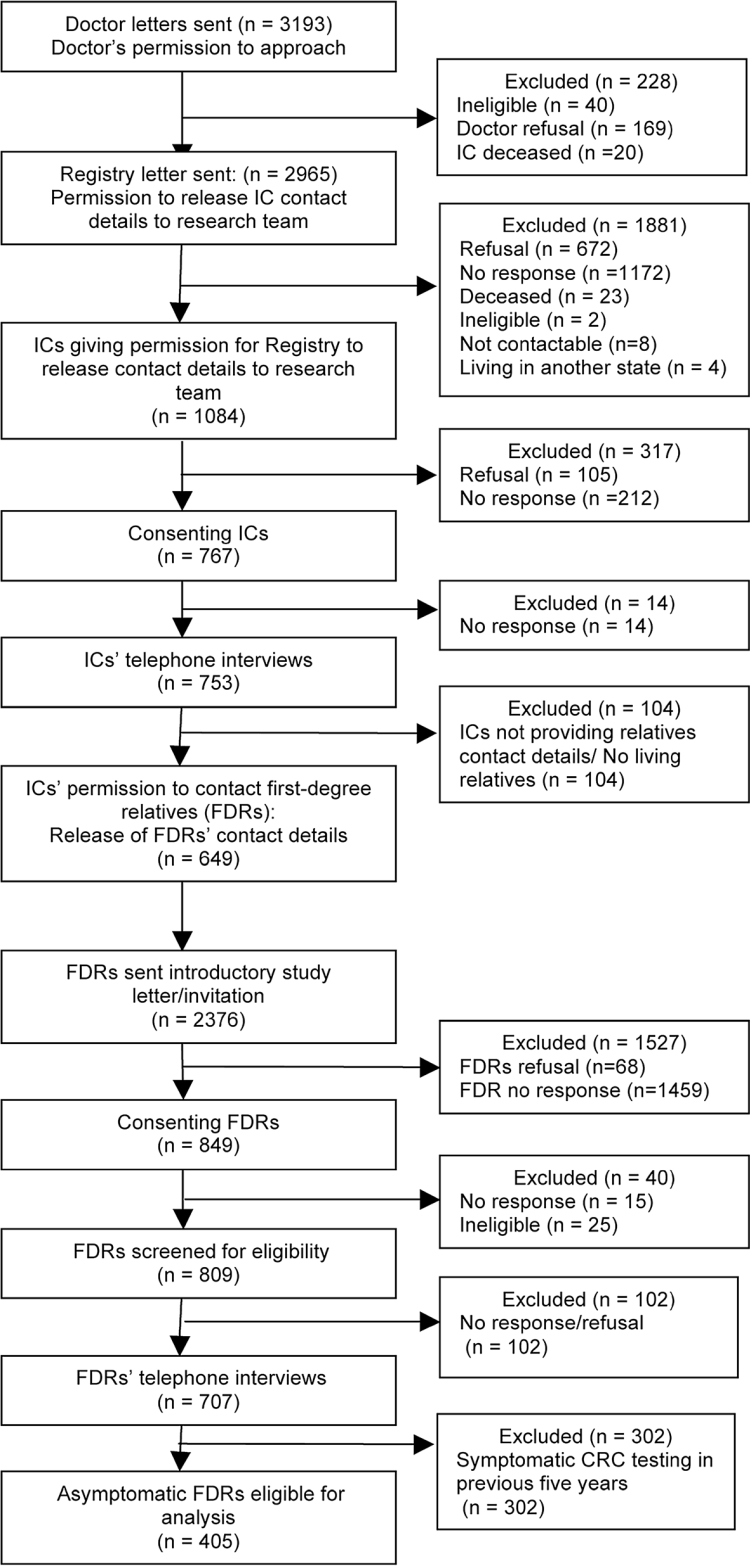
Flowchart representing selection and recruitment of asymptomatic first-degree relatives of colorectal cancer patients.

Index cases (ICs) aged 18 years or older, within 9 months of CRC diagnosis, registered with the VCR and English-speaking were eligible to participate in this study. The VCR checked the Familial Adenomatous Polyposis (FAP) Register to exclude persons with FAP. The VCR wrote to the clinicians of eligible patients to advise them of the study and request consent to approach the index cases. Clinicians were asked to notify the VCR if there was any reason why a person should not be invited to participate in the study. Patients for whom the treating clinicians did now allow consent to patient approach from the VCR were not contacted. The VCR wrote to the remaining patients seeking permission to release their contact details to the research team. Index cases who agreed to provision of their contact details to the researchers were contacted by the research team *via* mail and asked to participate in the study. To accommodate index case preferences about the mode of approach to their relative, consenting index cases provided the details of FDRs aged 18 years or older for the purposes of contacting them *via* either (i) the research team (with their permission) who sent a study invitation by post on their behalf or (ii) a study invitation mailed to the patient who passed this invitation on to the relative(s). Eligibility criteria for FDRs’ participation were (1) English-speaking, aged 18 years or older, and (2) no previous history of advanced adenoma, CRC, ulcerative colitis, Crohn’s disease, inflammatory bowel disease or FAP. First-degree relatives meeting these criteria were eligible to complete the baseline telephone interviews. Assessment of first-degree relatives CRC screening behaviour occurred at approximately nine to twelve months post index cases diagnosis, however, this could have fluctuated dependent on the time taken to recruit the index case or their first-degree relatives into the study. They were classified as asymptomatic and eligible for CRC screening if they had not undertaken FOBT, sigmoidoscopy or colonoscopy due to a symptom episode in the previous five years. A diagrammatic representation of the recruitment protocol is presented in Figure 1.

### Quantifying risk based on family history of colorectal cancer

Index cases were asked about family history of CRC, including all first- and second-degree relatives and their ages at diagnosis, if relevant. Index cases’ ages at diagnosis were obtained from VCR data. The FDRs of index cases were allocated to a level of risk in accordance with screening guidelines (See Table 1).

**Table 1:** Description of risk categories and their respective screening recommendations in accordance with National Health and Medical Research Council colorectal cancer screening guidelines

**Risk category**	**Risk features**	**Screening recommendation**
At or slightly above average risk	·No personal history of bowel cancer	FOBT every second year from the age of 50 years.
	·Either no close relatives with bowel cancer or one first-degree or second-degree relative with bowel cancer diagnosed at age 55 years or older.	
		Consider sigmoidoscopy (preferably flexible) every five years.
Moderately increased risk	·One first-degree relative diagnosed before the age of 55 years (without potentially high-risk features listed below), or	Colonoscopy every five years starting at age 50, or at an age 10 years younger than the age of first diagnosis of CRC in the family, whichever comes first.
	·Two first-degree relatives or one first- and one second-degree relative(s) on the same side of the family (without potentially high-risk features listed below).	
Potentially high risk	·Three or more first-degree or a combination of first-degree and second-degree relatives on the same side of the family diagnosed with bowel cancer (suspected HNPCC*), or	Dependent on presence and type of familial cancer.
		At least colonoscopy every 5 years.
		Age of screening commencement dependent on familial colorectal cancer syndrome identified***
	·Two or more first-degree or second-degree relatives on the same side of the family diagnosed with bowel cancer, including any of the following high-risk features:	
	- bowel cancer before the age of 50 years	
	- multiple bowel cancers in the one person	
	- at least one relative with cancer of the endometrium, ovary, stomach, small bowel, renal pelvis, ureter, biliary tract or brain	
	- at least one first-degree relative with a large number of adenomas throughout the large bowel (suspected FAP)**	
	- somebody in the family in whom the presence of a high-risk mutation in the adenomatous polyposis coli (APC) gene or one of the mismatch repair (MMR) genes has been identified.	

*HNPCC: Hereditary non-polyposis colorectal cancer or Lynch’s Syndrome. ** FAP: Familial Adenomatous Polyposis. *** See guidelines for syndrome specific CRC screening recommendation.

### Colorectal cancer screening history

FDRs were asked separately whether they had ever undertaken any of the following CRC tests: FOBT; sigmoidoscopy; or colonoscopy. Respondents indicating “Yes” to any of these tests were asked to specify how long ago their most recent test was undertaken and the reason for the test, to establish whether the respondent was asymptomatic at the time of testing.

### Eligibility for screening

Asymptomatic FDR respondents “at or slightly above average risk” were eligible for screening if they were aged 50 years or older. For respondents at “moderately increased risk”, in accordance with guidelines, eligibility for CRC screening was determined on the basis of “starting at age 50 years or at an age 10 years younger than the age of first diagnosis of bowel cancer in the family, whichever comes first”. Asymptomatic respondents at “potentially high risk” were eligible for screening if they were aged 18 years or older.

### Statistical analysis

The proportions of FDRs ever receiving CRC testing (i.e. FOBT, sigmoidoscopy or colonoscopy) overall and by level of risk (i.e. “at or slightly above average risk”, “moderately increased risk” and “potentially high risk”) were calculated by the number of FDRs reporting receiving any CRC test, divided by the total number of FDRs. The proportion of FDRs screened in accordance with Australian screening guidelines [18] was assessed according to level of risk as follows: “at or slightly above average risk” (FOBT every two years, consider sigmoidoscopy, preferably flexible, every five years); and “moderately increased risk” or “potentially high risk” (colonoscopy every five years). Over-screening was not assessed in this study as information was only obtained on the most recent testing for each test type. CRC screening undertaken before index case CRC diagnoses was included in the analysis. Associations between ever-tested and guideline-recommended screening were explored for the following items: socio-demographic characteristics (i.e. age, gender, education, marital status, Australian born, employment situation, private health insurance); geographical location (Accessibility/Remoteness Index of Australia); relationship to index case (parent, child, sibling); quality of life (Euro-Qol EQ-5D, VAS score);[32] worry about bowel cancer (Worried/Not worried); and ever asked about family history of bowel cancer by doctor/health professional (Yes/No). Logistic regression modelling in a generalised estimation equation framework was used to adjust for multiple FDRs within families. Simple associations were examined first before all covariates were added to a multiple logistic regression model. Variables with *p-*value <.10 were retained in the final model. Parents of index cases were excluded from multiple regression models in all analyses due to the small number of parents recruited in the sample.

## Results

Of the 748 index cases interviewed, 98% had living FDRs. Of the index cases with living FDR(s), 88% gave consent for the research team to send a study invitation to at least one FDR. A total of 2376 study invitations were sent to FDRs, with 707 (30%) FDRs participating in the study. A total of 405 FDRs were deemed asymptomatic and eligible for analysis. The proportions of asymptomatic FDRs recruited per family were as follows: 56% (107) of families had one FDR recruited; 23% (43) had two FDRs recruited; 15% (29) had three FDRs recruited; and 6.3% (12) had more than three FDRs recruited. Table 2 describes the characteristics of the asymptomatic study population.

**Table 2:** Characteristics of asymptomatic first-degree relatives of colorectal cancer patients (n = 405)

**Characteristic**	**n**	%*
Risk category		
At or slightly above average risk	166	41
Moderately increased risk	43	11
Potentially high risk	195	48
Sex		
Male	165	41
Female	240	59
Highest level of education		
University degree	164	41
Trade or TAFE Certificate/Diploma	72	18
Secondary schooling completed	44	11
Secondary schooling not completed	123	31
Marital status		
Married/partnered	315	78
Not partnered	90	22
Born in Australia		
No	33	8
Yes	372	92
Employment situation		
Full-time	172	42
Part-time	87	21
Not working	114	28
Other	32	8
Private health insurance		
No	122	30
Yes	283	70
Geographical location (ARIA)		
Major cities (urban)	211	54
Regional/remote	182	46
Relationship to index case		
Parent**	22	5
Sibling	212	52
Child	171	42
Worry about bowel cancer		
Not worried	175	43
Worried	230	57
Ever asked about family history of bowel cancer by doctor/health professional		
No	258	64
Yes	147	36
	Mean	SD
Age (years)	56.5	13
Quality of life (EQ-5D VAS score)	80.8	13

* Percentage of responses (excluding any missing values). **Parents removed from further regression analyses due to small cell size (n = 22).

### Ever received colorectal cancer testing

Overall, 69% (278/405) of FDRs had ever received any CRC testing (i.e. FOBT, sigmoidoscopy or colonoscopy) in their lifetime. The proportions of FDRs ever undertaking CRC testing within each risk category were as follows: “at or slightly above average risk” (70%, 116/166); “moderately increased risk” (70%, 30/43); and “potentially high risk” (67%, 131/195). No significant differences in CRC testing across risk category were identified (χ^2^ = 0.3, *df* = 2, *p* = .86). Multiple logistic regression analyses (see Table 3) revealed that older FDRs, those with private health insurance, siblings of the index case and FDRs who had ever discussed family history of CRC with a doctor were at significantly greater odds of ever receiving CRC testing.

**Table 3:** Multiple logistic regression analysis of factors associated with ever receiving colorectal cancer testing

	N (%)*	**OR (95% CI)**	***p *value**
Private health insurance			
Yes	201 (71)	2.05 (1.26, 3.33)	0.0039
No	77 (63)	1	
Relationship to index case			
Sibling	166 (78)	2.19 (1.32, 3.63)	
Child	92 (54)	1	0.0024
Ever asked about family history of bowel cancer by doctor/health professional			
Yes	125 (85)	4.78 (2.80, 8.18)	<.0001
No	153 (59)	1	
	Mean (SD)	OR (95% CI)	*p* value
Age			
Yes	58.8 (11.01)	1.04 (1.02, 1.07)	0.0002
No	51.3 (15.64)	1	

* Percentage of responses (excluding any missing values).

### Colorectal cancer screening in accordance with guideline recommendations

#### First-degree relatives “at or slightly above average risk”

**Figure 2:**
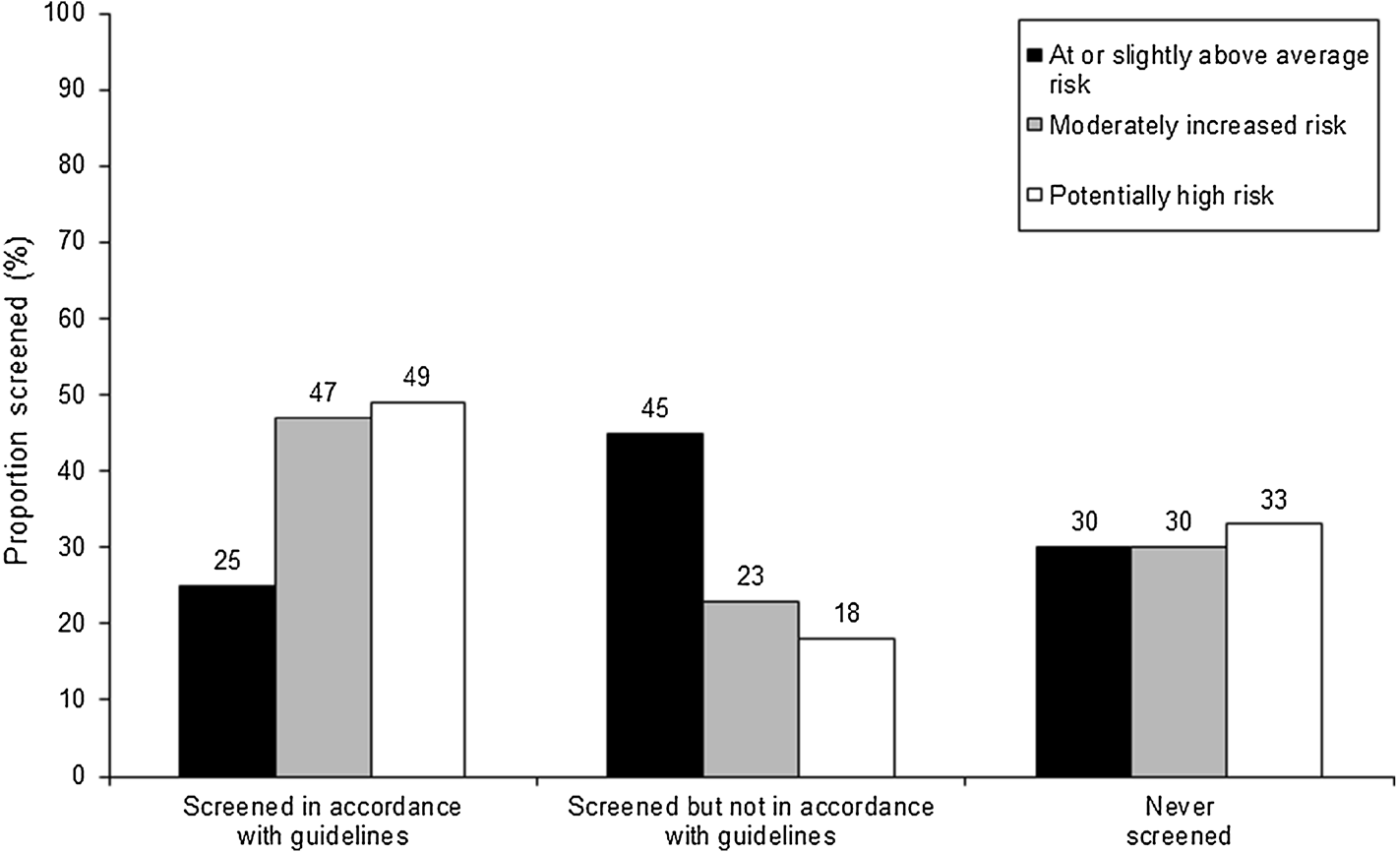
First-degree relatives’ colorectal cancer screening status in accordance with guidelines by level of risk.

Of the 166 FDRs “at or slightly above average risk”, 25% (42/166) were screened in accordance with NHMRC screening guideline recommendation (See Figure 2). All respondents screened in accordance with guidelines had undertaken FOBT screening. Thirty percent (50/166) of persons “at or slightly above average risk” had never undertaken any CRC testing in their lifetime. The remaining persons “at or slightly above average risk” (45%, 74/166) had either undertaken FOBT screening outside the recommended guideline timeframe (i.e. every two years) or had undertaken colonoscopy screening, a test type not endorsed in screening guidelines for persons “at or slightly above average risk”. For persons “at or slightly above average risk”, the number of colonoscopies resulting from positive FOBT was not obtainable, although the number of such cases is likely to be small, as 59% (37/63) of respondents “at or slightly above average risk” who had undertaken colonoscopy screening in the previous five years had previously never undertaken guideline-recommended FOBT or sigmoidoscopy testing.

#### First-degree relatives at “moderately increased risk”

Of the 43 respondents at “moderately increased risk”, 47% (20/43) were screened in accordance with NHMRC screening recommendation (i.e. colonoscopy screening every five years). Thirty percent (13/43) of respondents had never undertaken any CRC testing (see Figure 2). Of the remaining 23% (10/43), half (5/10) had undertaken FOBT screening in the previous two years.

#### First-degree relatives at “potentially high risk”

Of the 195 respondents at “potentially high risk”, 49% (95/195) were screened in accordance with NHMRC screening recommendation (i.e. colonoscopy screening every five years). Thirty-three percent (64/195) of respondents had never undertaken any CRC testing. The remaining 18% (36/195) of respondents had undertaken CRC screening not in accordance with guideline recommendation (see Figure 2). Of these, 61% (22/36) had undertaken FOBT screening in the previous two years.

### Factors associated with screening in accordance with guidelines

Multiple logistic regression models for factors associated with screening guidelines across level of risk are presented in Table 4. FDRs “at or slightly above average risk” with higher levels of education were significantly more likely to be screened in accordance with guideline recommendations, compared with FDRs with lower levels of education. Further, male FDRs “at or slightly above average risk” were significantly more likely to be screened in accordance with guidelines, compared with female FDRs. Due to the small number of respondents in the “moderately increased risk” group (n = 43), both “moderately increased risk” and “potentially high risk” groups were combined in the analysis of screening in accordance with screening guidelines. Persons at “moderately increased risk” and “potentially high risk” who were married or partnered, living in a major city or urban area, sibling of the index case and ever asked about family history of CRC by a doctor were significantly more likely to be screened in accordance with guideline recommendations.

**Table 4:** Multiple logistic regression model of factors associated with first-degree relatives’ screening in accordance with guidelines

**“At or slightly above average risk”**	N (%)*	**OR (95% CI)**	***p *value**
Gender			
Male	27 (35)	2.74 (1.32, 5.68)	.0068
Female	15 (17)	1	
Highest level of education			
Secondary schooling completed	3 (19)	0.34 (0.09, 1.20)	0.0941
Secondary schooling not completed	10 (18)	0.41 (0.18, 0.91)	0.0288
Trade or TAFE Certificate/Diploma	4 (14)	0.21 (0.07, 0.65)	0.0070
University degree	25 (38)	1	
“Moderately increased risk” and “Potentially high risk”	N (%)	OR (95% CI)	*p* value
Marital status			
Married/partnered	97 (55)	3.68 (1.72, 7.88)	0.0008
Not partnered	18 (30)	1	
Geographical location (ARIA)			
Major cities (urban)	68 (56)	2.26 (1.27, 4.03)	0.0056
Regional/remote	41 (39)	1	
Relationship to index case			
Sibling	74 (62)	5.15 (2.28, 11.67)	0.0001
Child	29 (30)	1	
Ever asked about family history of bowel cancer by doctor/health professional			
Yes	65 (71)	5.08 (2.55, 10.11)	.0001
No	50 (34)	1	

* Percentage of responses (excluding any missing values).

## Discussion

Rates of ever receiving CRC testing for FDRs of CRC patients were relatively high in the current study, with approximately 70% of FDRs across each level of risk ever receiving CRC testing. The rate of FDRs ever receiving CRC testing was not found to be significantly higher among population groups at higher relative risk of CRC (i.e. persons at “moderately increased risk” and “potentially high risk”, compared with persons “at or slightly above average risk”). In an Australian context, rates of participation in CRC testing or testing undertaken in adherence to guidelines among FDRs of CRC have previously only been evaluated in two other studies [28,31].

The current study highlighted low levels of CRC screening in accordance with guideline recommendations across varying levels of risk: “at or slightly above average risk” (25%); “moderately increased risk” (47%); and “potentially high risk” (49%). International comparisons of risk-appropriate screening in accordance with guideline recommendations are difficult to ascertain, given that healthcare authorities’ endorsement of screening modality and timing of repeat testing vary across countries [18,33,34]. Few studies have assessed CRC screening in accordance with guideline recommendations for persons with an affected FDR with CRC [28-30]. This study, to our knowledge, is the first Australian population-based examination of CRC screening participation among FDRs of CRC patients

### Screening of first-degree relatives “at or slightly above average risk” in accordance with guidelines

The low rate of screening in accordance with guideline recommendation for FDRs “at or slightly above average risk” identified in this study is comparable to that of the general population in Australia [35,36]. Two separate population-based evaluations among persons over 50 years of age have indicated that 33% of respondents at “average risk” had undertaken FOBT screening in the previous five years [36] and 18.4% in the previous two years [35]. The most recent investigation of CRC screening participation among at-risk persons (i.e. those aged over 55 years) since the National Bowel Cancer Screening Program’s introduction in 2006 indicated that screening in accordance with guideline recommendation for persons “at or slightly above average risk” was 20 per cent [24]. Although community-based studies in the United States (US) have generally established rates of FOBT screening in accordance with guideline recommendations among FDRs of CRC patients of between 9% and 42%, [37-40] the studies with the highest screening rates had recruited participants through advertisements [38,39]. Such samples are unlikely to be representative of FDRs of CRC patients in general, and are likely to be biased as they include participants more likely to engage in screening, [23] thus reducing the findings’ relevance for indicating population-based estimates.

### Screening of first-degree relatives at elevated risk (“moderately increased risk” and “potentially high risk”) in accordance with guidelines

Current study findings indicated that 47% of FDRs of CRC patients at “moderately increased risk” and 49% of persons at “potentially high risk” were screened in accordance with the Australian guideline recommendation (i.e. colonoscopy screening every five years). This is much higher than other Australian data on risk-appropriate CRC testing among FDRs, which had identified three FDRs among 225 were screened in accordance with Australian guidelines [28]. The most recent study in Victoria among first and second-degree relatives selected from case and population control probands in the Melbourne metropolitan area identified that 6% (70/1236) of persons at “moderately increased risk” were screened in accordance with guideline-recommendation [31]. For relatives of probands at “potentially high risk” 1% (3/389) were adherent to guideline- recommended screening [31].

For the most part, the rate of screening in accordance with guideline recommendations for FDRs of CRC patients at elevated levels of risk (i.e. “moderately increased risk” and “potentially high risk”) in the current study is similar to rates identified in the general population [24,35]. The most recent Australian community-based study of CRC screening participation found that among persons over 55 years of age, 45% of those at “moderately increased risk” and “potentially high risk” were screened in accordance with guideline CRC screening recommendation (i.e. colonoscopy screening every five years) [24]. Another population-based investigation among persons aged over 50 years at “above average risk” indicated that 30% had undertaken endoscopy (colonoscopy or sigmoidoscopy) in the previous five years [35]. Data available from a US population-based National Health Interview Survey sample of persons 41-75 years of age with a FDR diagnosed with CRC indicated that 28% of FDRs had undertaken colonoscopy screening in the previous ten years [21]. For this study, direct compliance with guideline screening recommendation was not ascertained. Another study recruiting siblings of CRC patients diagnosed younger than 56 years of age through four cancer centres in the US indicated that 57% of siblings older than 34 years were screened in accordance with guidelines [29]. Study findings from another investigation of FDRs of CRC patients participating in a free FOBT screening program indicated that 22% of respondents were screened in accordance with guidelines [30]. In Canada, the most recent evaluation of CRC screening among FDRs of CRC patients aged over 40 years selected through the Alberta Cancer Registry indicated that 60% were appropriately screened for CRC screening (i.e. FOBT within one year, barium enema or sigmoidoscopy within five years, or colonoscopy within 10 years) [41]. In summary, the available evidence suggests that, worldwide, the screening rates of FDRs of CRC patients in accordance with guideline recommendations rarely exceed 50 percent, well short of rates likely to be necessary for reducing CRC incidence and mortality on a population basis.

### Factors associated with colorectal cancer testing and adherence to colorectal cancer screening guidelines

The present study identified a number of socio-demographic and provider-level factors impacting upon CRC testing and screening in accordance with guideline recommendations. It is well-established that older age is a consistent predictor of CRC screening [23,42,43]. The current study identified that the odds of receiving CRC testing increased with increasing age of FDRs. This finding is consistent with previous literature among the FDR population [25,41,44]. For persons “at or slightly above average risk”, adherence to screening guidelines was significantly more likely to occur for male compared with female FDRs. This is contrary to other studies of FDRs that have, on the whole, largely indicated no association between gender and screening behaviour [23].

Previous literature has indicated that having medical insurance is significantly correlated with CRC screening adherence [36,43,45]. Consistent with this literature, the current study identified that the likelihood of ever receiving CRC testing was at significantly higher odds for FDRs with private health insurance. This suggests that the costs of medical consultation or screening itself represent significant barriers to CRC screening rates among persons without private health insurance.

The current study also found that siblings compared with children of the index case were at significantly increased likelihood of ever receiving CRC testing and receiving CRC screening in accordance with screening guideline recommendations for persons at “moderately increased risk” and “potentially high risk”. Previous literature has largely analysed either CRC screening among siblings only [29,46] or FDRs combined and not separated into type of FDRs (i.e. parent, child or sibling) [37,41,47]. A previous Australian investigation found that being a sibling of a CRC patient, consistent with current study findings, was significantly associated with increased likelihood of previous participation in CRC testing [28]. Further, a multi-centre nation-wide study in Spain identified a higher rate of adherence to colonoscopy screening for siblings and children, compared with parents, when offered screening by a gastroenterologist [26]. Current data suggest that screening compliance may be lower among CRC patients’ children who are deemed eligible for screening in accordance with screening guidelines compared to their siblings. There is a pressing need to ensure equality in CRC screening uptake across at-risk FDRs e.g. children, siblings and parents.

This study also found that persons “at or slightly above average risk” with a higher level of education were at significantly increased odds of receiving screening in accordance with guideline recommendations. Previous findings related to CRC testing and educational attainment for persons with a family history of CRC are mixed, with some studies indicating a positive trend [29,30,48] and others indicating no association between CRC testing and level of education [28,38,47]. For persons at “moderately increased risk” and “potentially high risk”, screening according to guideline recommendations was significantly more likely to occur for FDRs who were married or partnered, compared with those not partnered. For the most part, marital status has been identified as a significant factor influencing screening participation in the general population [43,49-51] and among FDRs of CRC patients, [21,47] with increased screening compliance commonly found for married and partnered persons.

The role of geographical barriers to CRC screening was also evident in the current study, as screening in accordance with guideline recommendations for persons at “moderately increased risk” and “potentially high risk” was significantly more likely to occur for persons residing in metropolitan areas, compared with regional or remote areas. This finding is plausible, given the high concentration of colonoscopy services in major hospitals in large cities in Australia [52]. Future attention to equality in access to CRC screening services for those at increased levels of risk (i.e. “moderately increased risk” and “potentially high risk”) is clearly required.

The FDRs of CRC patients in the current study who had ever been asked about their family history of CRC by a doctor were also at significantly higher odds of ever undertaking CRC testing and undertaking testing in accordance with guideline recommendations for persons at “moderately increased risk” and “potentially high risk”). This finding highlights the need for assessment and on-going monitoring of family history of CRC within both the primary and specialist healthcare settings. Although physicians have been found to follow appropriate guideline recommendations for CRC screening once increased risk has been identified, the family history information gathered is often insufficient for risk stratification purposes [53-55]. The wider incorporation of recently developed cancer risk assessment tools, showing both feasibility and effectiveness in the collection of family history information, automation of familial risk stratification and risk-appropriate screening advice, [56,57] should be considered in the primary and secondary healthcare systems.

Despite the evidence that physician recommendation to screen has a powerful motivating effect on CRC screening uptake, [58] FDRs are most often not informed by physicians about the need for CRC screening [59]. A significantly higher rate of colonoscopy screening can result among those siblings where the index cases were aware of their FDRs’ increased risk [27]. Improvements in CRC screening among the higher-risk populations (i.e. persons at “moderately increased risk” and “potentially high risk”) rely on physicians’ active involvement in discussions with index cases and their FDRs surrounding their families’ increased risk and need for CRC screening.

### Strengths and limitations of the study

In interpreting study findings, several limitations should be considered. The response rates among index cases and FDRs were low. Given that people interested in their health are more likely to participate in health research, it is likely that this has resulted in an over-estimate rather than an under-estimate of the true screening rates. It should be noted that the index case response rate achieved in the current study is comparable to the only other Australian-based investigation of CRC screening among FDRs that had adopted a Cancer Registry based recruitment method [28].

Personal history of adenomatous polyps was not investigated in this study, making it difficult to calculate precisely the proportion of persons “at or slightly above average risk” undertaking colonoscopy screening (i.e. in the previous five years) not in accordance with guideline recommendation. Nonetheless, the available data are suggestive of significant over-use of colonoscopy, given that a large proportion of persons “at or slightly above average risk” had received recent colonoscopy without any other CRC screening (i.e. FOBT or sigmoidoscopy) beforehand.

Family history and CRC screening behaviour were self-reported, rather than objectively assessed. Nonetheless, studies have indicated that self-reported family history of CRC is moderately accurate [60,63]. The level of risk obtained for FDRs was allocated on the basis of index case self-reported information about family history, thus allowing only a potential estimate. It was not practical to obtain a full family history from each FDR of CRC patients for this study.

## Conclusions

In summary, the current study identified a significant level of under-screening among a high-risk population and a substantial level of colonoscopy screening not in accordance with screening guidelines for persons “at or slightly above average risk”. There is an urgent need for enhanced physician and patient education about risk-appropriate screening for FDRs of CRC patients, and for further descriptive research to identify the barriers to CRC screening among this population group. Effective systematic interventions on a population basis are required to improve CRC screening participation of FDRs of CRC patients.

### Ethical approval

This study was approved by the University of Newcastle (No 2008-0047) and Cancer Council Victoria (No 0810) ethics committee, and all participants provided written consent.

## Competing interests

All authors have completed the Unified Competing Interest form at http://www.icmje.org/coi_disclosure.pdf (available on request from RJC) and declare that; (2) [RJC, CLP, RWSF, MLC, FAM, CD, DH, DB JS] have no relationships with any companies that might have an interest in the submitted work in the previous 3 years; (3) their spouses, partners, or children have no financial relationships that may be relevant to the submitted work; and (4) [RJC, CLP, RWSF, MLC, FAM, CD, DH, DB JS] have no non-financial interests that may be relevant to the submitted work.

Conflict of Interest Disclosure: No potential conflicts of interest.

## Authors’ contribution

All authors participated in the acquisition and analysis of data and critical revision of the manuscript, have seen and approved the final version, had full access to study data, and were jointly responsible for the decision to submit for publication. RJC is guarantor for the paper.

**Grant support:** This research was funded by a grant from the National Health and Medical Research Council (ID: 510776) and received infrastructure support from the Hunter Medical Research Institute. The Funders had no role in the design, implementation, analysis or preparation of the final manuscript. All researchers were independent from the funding source.

## Authors’ information

**Data sharing:** All authors had full access to data and take full responsibility for the integrity and accuracy of analysis. The dataset is available from Ryan Courtney at r.courtney@unsw.edu.au.

## Pre-publication history

The pre-publication history for this paper can be accessed here:

http://www.biomedcentral.com/1471-2407/13/13/prepub
